# Surveying the genome and constructing a high-density genetic map of napiergrass (*Cenchrus purpureus* Schumach)

**DOI:** 10.1038/s41598-018-32674-x

**Published:** 2018-09-26

**Authors:** Dev Paudel, Baskaran Kannan, Xiping Yang, Karen Harris-Shultz, Mahendar Thudi, Rajeev K. Varshney, Fredy Altpeter, Jianping Wang

**Affiliations:** 1 Agronomy Department, IFAS, University of Florida, Gainesville, FL 32611 USA; 2 Crop Genetics and Breeding Research Unit, USDA-Agricultural Research Service, 115 Coastal Way, Tifton, GA 31793 USA; 3 Center of Excellence in Genomics & Systems Biology, International Crops Research Institute for the Semi-Arid Tropics (ICRISAT), Hyderabad, 502324 Telangana State India; 4 Plant Molecular and Cellular Biology Program, Genetic Institute, University of Florida, Gainesville, FL 32611 USA; 5 Center for Genomics and Biotechnology, Key Laboratory of Genetics, Breeding and Multiple Utilization of Corps, Ministry of Education, Fujian Provincial Key Laboratory of Haixia Applied Plant Systems Biology, Fujian Agriculture and Forestry University, Fuzhou, Fujian 350002 China

## Abstract

Napiergrass (*Cenchrus purpureus* Schumach) is a tropical forage grass and a promising lignocellulosic biofuel feedstock due to its high biomass yield, persistence, and nutritive value. However, its utilization for breeding has lagged behind other crops due to limited genetic and genomic resources. In this study, next-generation sequencing was first used to survey the genome of napiergrass. Napiergrass sequences displayed high synteny to the pearl millet genome and showed expansions in the pearl millet genome along with genomic rearrangements between the two genomes. An average repeat content of 27.5% was observed in napiergrass including 5,339 simple sequence repeats (SSRs). Furthermore, to construct a high-density genetic map of napiergrass, genotyping-by-sequencing (GBS) was employed in a bi-parental population of 185 F_1_ hybrids. A total of 512 million high quality reads were generated and 287,093 SNPs were called by using multiple *de-novo* and reference-based SNP callers. Single dose SNPs were used to construct the first high-density linkage map that resulted in 1,913 SNPs mapped to 14 linkage groups, spanning a length of 1,410 cM and a density of 1 marker per 0.73 cM. This map can be used for many further genetic and genomic studies in napiergrass and related species.

## Introduction

Napiergrass (*Cenchrus purpureus* Schumach., syn. *Pennisetum purpureum* Schumach), also known as elephant grass, is a tropical perennial grass native to eastern and central Africa. It is cultivated primarily for forage and widely used by smallholder dairy farmers due to its high growth rate, leaf nutritive value, perennial nature, persistence, ease of propagation, and broad adaptation^1–4^. As a C_4_ grass species, napiergrass is a promising candidate feedstock for biofuel production due to its superior yield of biomass^5–7^. Napiergrass cultivars are typically developed from natural out-crossings^1,8^. It is an allotetraploid (2*n* = 4*x* *=* 28, A’A’BB)^9^ with an average amount of DNA per G_1_ nucleus of 5.78 pg^10^. The chromosomes in the A’ genome of napiergrass are believed to be homologous to the A genome of pearl millet (*Pennisetum glaucum*, 2*n* = 2*x* = 14, AA)^9^. Pearl millet and napiergrass form a monophyletic group^11^ and were initially classified as primary and secondary gene pool of the genus *Pennisetum*, respectively^11,12^. Recently, species of *Pennisetum* and *Odontelytrum* were transferred to the unified genus *Cenchrus*^4^. Pearl millet and napiergrass can hybridize to produce hybrids called kinggrass^13^ or Pearl Millet-Napiergrass (PMN) hybrids^14–16^. These hybrids are sterile due to triploidy (2*n* = 3*x* = 21, AA’B genome)^17^, thus preventing the unintended spreading into natural areas or other cropping systems by wind dispersed seeds. Some PMN hybrids show high heterosis for biomass yield and forage quality while the perennial, persistent nature is often reduced compared to napiergrass^3^.

The targeted improvement of napiergrass includes identification of agronomically superior genotypes and studies assessing genetic diversity and relatedness using random amplification of polymorphic DNA (RAPD), amplified fragment length polymorphism (AFLP), isozymes, and simple sequence repeats (SSRs) developed for other species like pearl millet and buffelgrass (*Pennisetum ciliare*)^1,13,18–23^. Other than these, genetic information on napiergrass is very meager^23^. A genetic map is lacking and molecular tools are not yet deployed in napiergrass breeding programs^21,24^. Development of molecular markers for detection and utilization of DNA polymorphisms will help to understand the molecular basis of various agronomic traits^25^. Molecular breeding for yield components, flowering date, nutrient uptake, abiotic and biotic stress tolerance will accelerate genetic improvement of napiergrass. This can be greatly facilitated by having access to marker resources like SSR, single nucleotide polymorphisms (SNPs), and genetic linkage maps. SSRs as molecular markers are very advantageous because they are locus specific, multi-allelic, co-dominant, and easy to detect by polymerase chain reaction (PCR)^26,27^. SNP markers have gained increasing consideration in molecular breeding and linkage map construction as they occur in a large number and high density^28^. Access to these resources will support marker-assisted selection (MAS) by making phenotypic predictions based on the genotype^29^.

Recently, next generation sequencing (NGS) technology has simplified linkage map construction by using high throughput genotyping-by-sequencing (GBS), which allows simultaneous SNP discovery and genotyping across the whole genome of the population of interest^29–31^. GBS has been effective for marker discovery, genetic mapping, quantitative trait locus (QTL) analysis, population genetics, and comparative genomics studies in several diploid species and has recently gained popularity in polyploid species such as wheat (*Triticum aestivum*)^32^, switchgrass (*Panicum virgatum*)^33^, potato (*Solanum tuberosum*)^34^, and sugarcane (*Saccharum* spp.)^35^ among others. However, the presence of highly similar homeologous copies of two genomes in allopolyploid species complicates SNP detection which relies on delineating true allelic SNPs from homeologous SNPs because sequences from homeologous loci mimic allelic SNPs and can introduce false-positives. Distinguishing allelic SNPs from homeologous SNPs relies on the use of high-stringency sequence read alignment, specifically uniquely aligned reads^36^. Despite challenges of using GBS for genotyping of polyploid species, genetic mapping without a reference genome has been carried out in switchgrass by defining linkage groups with the modulated modularity clustering (MMC) method^37^ referring to the genome of foxtail millet (*Setaria italica*)^33^. A genetic map of wheat was constructed by using the bin-mapping procedure with homozygous genotypes of a double-haploid population^38^. Each program for calling variants utilizes different models or algorithms to identify potential polymorphisms, therefore, multiple software programs need to be evaluated in order to identify the best SNP caller for polyploids^36^.

Linkage maps are important tools for map-based cloning, marker-assisted breeding, QTL identification, genome organization, and comparative genomics of important species. A number of linkage maps have been constructed for several grasses including pearl millet^39^. However, so far, napiergrass SSR markers, genetic linkage map, or reference genome assembly are lacking. The purpose of this study was to survey the napiergrass genome and to construct a high-density genetic linkage map. Here, for the first time, we have surveyed whole genome sequences in napiergrass, developed SSR markers, and constructed high-density genetic map of napiergrass to investigate its genomic and genetic architecture.

## Results

### Napiergrass genome survey

Approximately 211 million raw reads from Illumina and 97 thousand raw reads from 454 sequencing were subjected to a sequence quality check. After filtration and trimming, 161 million clean Illumina reads and 96,000 clean 454 reads were aligned to the pearl millet genome v1^40^. A total of 62.5 million (38.8%) reads were able to align with the pearl millet genome. Polymorphisms were detected between the napiergrass and pearl millet aligned reads, of which 619,708 SNPs and 24,135 indels were identified. Most of the sequence variations (58.7% SNPs) were in intergenic regions (Supplementary Fig. S1). The clean reads were assembled into 113,789 contigs with a total size of 44.5 Mbp and a N50 of 435 bp and a GC content 43.45%. The largest 10 contigs of the sequence assembly, which ranged from 8,506 to 25,329 bp in length, were selected as representative napiergrass genome fragments. The repeat content of the 10 longest contigs ranged from 5% to 90% with an average of 27.5% and a total of 164 repetitive elements (Supplementary Table S1). Two contigs had no hits in the pearl millet genome due to a high repeat content (over 80%). The rest of the contigs had one or more large hits (>500 bp) to the pearl millet genome. The micro-synteny showed mostly collinear relationship between the genomes of the two species (Supplementary Fig. S2). However, chromosome inverted duplications were also observed in the pearl millet genome (Supplementary Fig. S3), indicating that the chromosome rearrangement occurred after the speciation of napiergrass and pearl millet. The length of stringently (>500 bp and >80% sequence similarity) aligned regions accounted for 36.3% of the examined contig sequences of napiergrass (Supplementary Table S2). The total length of the alignment was 25.1% higher in pearl millet than in napiergrass aligned regions.

From the assembled napiergrass survey sequences, 5,339 SSRs were identified. Mono- type repeats were most common in napiergrass, followed by Tri-, Di- and Tetra- type repeats (Supplementary Table S3). From these identified SSRs, 1,926 were successfully used for primer design (Supplementary Table S4). All of the primer sequences aligned to the assembly of napiergrass and 89% of the primers were uniquely aligned. On the other hand, the overall alignment rate of the primers with pearl millet genome v1^40^ was 31% with 15% uniquely aligned. These SSR primers will undoubtedly serve as an abundant resource for molecular markers in napiergrass.

### Genotyping-by-sequencing

To construct the linkage map for napiergrass, an F_1_ bi-parental mapping population was developed, which consisted of 185 true hybrid individuals^41^. These hybrids were subjected to GBS. A total of 549 million raw reads were generated. After trimming and filtering, 512 million high quality reads were retained. The average number of reads per sample was 2.6 million and ranged from 44 thousand to 5.4 million reads per sample. *In silico* digestion of the pearl millet genome v1^40^ with *Pst*I yielded DNA fragments in the range of 170–350 bp, which suggest that an estimated average depth for the mapping population was 36.5 reads per locus per sample (Supplementary Fig. S4), which should allow us to call the SNPs confidently at most of the loci.

A total of 695,602 unique tags were identified from the clean reads generated from the mapping population by using the TASSEL *de-novo* UNEAK pipeline. These tags were further clustered into 182,934 non-redundant tags by CD-HIT. To examine the sequence similarity between napiergrass and other grass species, we aligned the non-redundant tags of napiergrass against several grass species with complete genome sequences including rice (*Oryza sativa*) (Osativa_323_v7.0), *Brachypodium* (Bdistachyon_314_v3.0), maize (Zmays_284_AGPv3), sorghum (Sbicolor_313_v3.0), foxtail millet (Sitalica_312_v2), switchgrass (Pvirgatum_273_v1.0), wheat (Taestivum_296_v2), pearl millet v1^40^, and barley (ASM32608v1), with *Arabidopsis* (Athaliana_167_TAIR9) as an outgroup control. The results showed that the percentage of napiergrass sequence tags aligned to these grass species ranged from 2.6% to 37.9% for barley and pearl millet genome, respectively (Table 1), indicating a relatively close relationship between napiergrass and pearl millet.

**Table 1:** Summary of the alignment of non-redundant tags of napiergrass (*Cenchrus purpureus*) to the available genomes of different species.

Genome used (species name)	Number of tags with blast hits	Percentage of tags with blast hits (%)
Arabidopsis (*Arabidopsis thaliana*)	120	0.07
Purple false brome (*Brachypodium distachyon*)	6,029	3.30
Barley (*Hordeum vulgare*)	4,751	2.60
Rice (*Oryza sativa*)	6,879	3.76
Pearl millet (*Pennisetum glaucum*)	69,385	37.93
Switchgrass (*Panicum virgatum*)	24,163	13.21
Sorghum (*Sorghum bicolor*)	14,654	8.01
Foxtail millet (*Setaria italica*)	40,849	22.33
Wheat (*Triticum aestivum*)	6,459	3.53
Maize (*Zea mays*)	11,972	6.54

### SNP calling by various SNP callers

**Figure 1:**
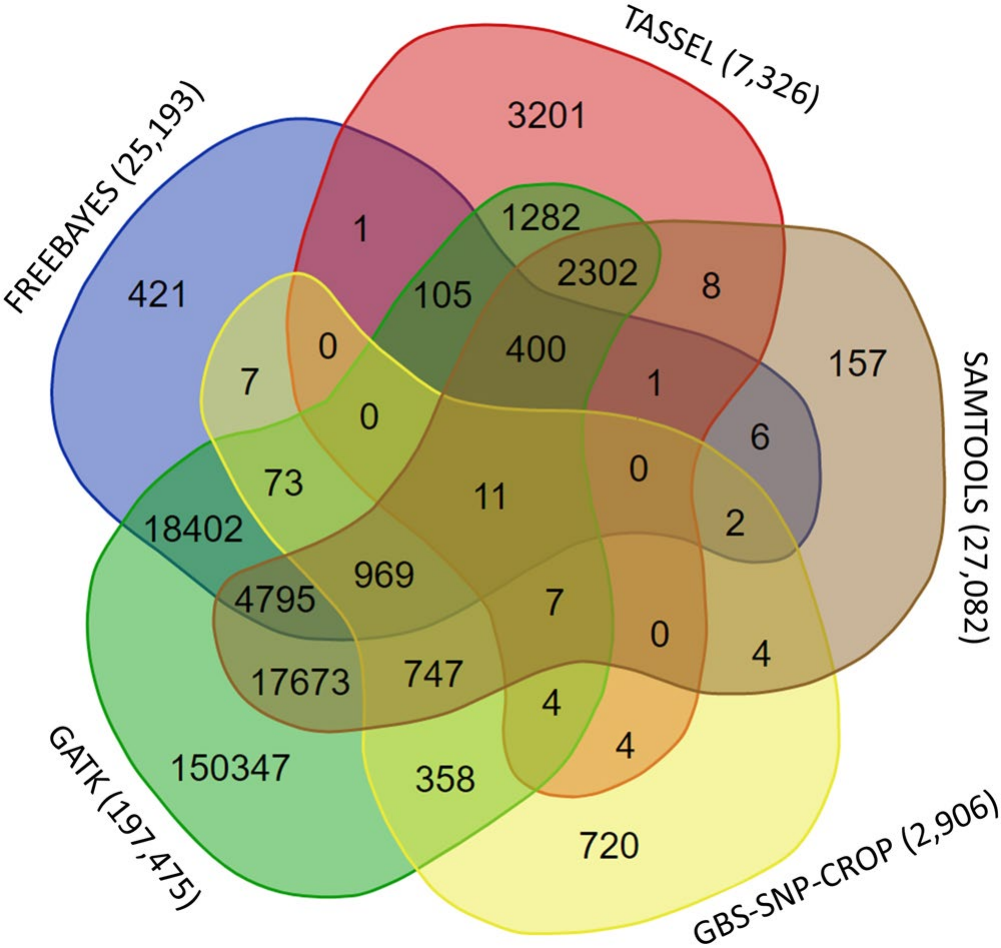
Venn diagram showing concordant napiergrass SNPs called by five reference-based SNP callers, SAMtools, GBS-SNP-CROP, GATK, FreeBayes, and TASSEL. Numbers in parenthesis after the program name shows the total number of SNPs called by each program.

Three *de-novo* SNP calling pipelines, TASSEL-UNEAK, Stacks, and GBS-SNP-CROP identified 10,799, 6,871, and 4,521 SNPs, respectively. Reference based pipelines were also applied by using pearl millet v1^40^ as the reference genome. However, the alignment rate was relatively low due to the differences between the napiergrass and pearl millet genomes. The percentage of clean reads aligned to the pearl millet genome using Bowtie2 ranged from 5.60% to 44.62% with an average of 39.68%. Two samples had a small number of sequences (<10% of the average number of sequences per sample) and also the lowest percentage of uniquely mapped reads (Supplementary Table S5, Supplementary Fig. S5). Therefore, these samples were removed from linkage map construction. Six different reference-based pipelines were employed to call SNPs viz., TASSEL 4.3^42^, Stacks 1.24^43^, GBS-SNP-CROP^44^, SAMtools 1.2 mpileup^45^, FreeBayes 0.9.21^46^, and GATK 3.3^47^. TASSEL 4.3, Stacks, and SAMtools identified 7,326, 4,920, 27,082 SNPs, respectively in the mapping population, whereas FreeBayes, GBS-SNP-CROP, and GATK that can handle ploidy identified 25,193, 2,906 and 197,475 SNPs, respectively. The six reference-based SNP callers concordantly called only 11 SNPs (Fig. 1, only five programs are shown in figure due to Venn-diagram display limitations) and 207,391 non-redundant SNPs.

### Genetic linkage map construction

From a total of 549,944 SNPs called by both reference based and *de-novo* pipelines, 287,093 SNPs were filtered for further analysis. Out of these, a total of 18,286 single-dose SNPs were genotyped in more than 180 progenies. Three individuals with more than 10% missing sites were removed from further analysis. For linkage map construction of each parental line, only the SNPs showing heterozygous in one parent but homozygous in the other parent were selected. A total of 3,276 loci were heterozygous in female parent but homozygous in male parent and segregated with an expected ratio of 1:1 in the population, thus can be used for female parent linkage map construction. Similarly, 3,417 loci were heterozygous in male parent but homozygous in female parent and segregated with an expected ratio of 1:1 in the population, thus can be used for male parent linkage map construction. For the female parental line, a total of 1,606 SNPs were grouped and 899 loci were mapped on 14 linkage groups with a total length of 1,555.17 cM averaging 1 marker every 1.72 cM (Supplementary Fig. S6). Inclusion of segregation distorted (SD) markers increased the genetic distance of the female parent map by 28.13%. For the male parent, a total of 1,509 markers were grouped into 14 linkage groups and 1,073 markers were mapped onto these 14 linkage groups with a total length of 1,939.19 cM averaging 1 marker every 1.80 cM (Supplementary Fig. S7). Inclusion of SD markers increased the total genetic distance of the male parent map by 38.41%.

**Figure 2:**
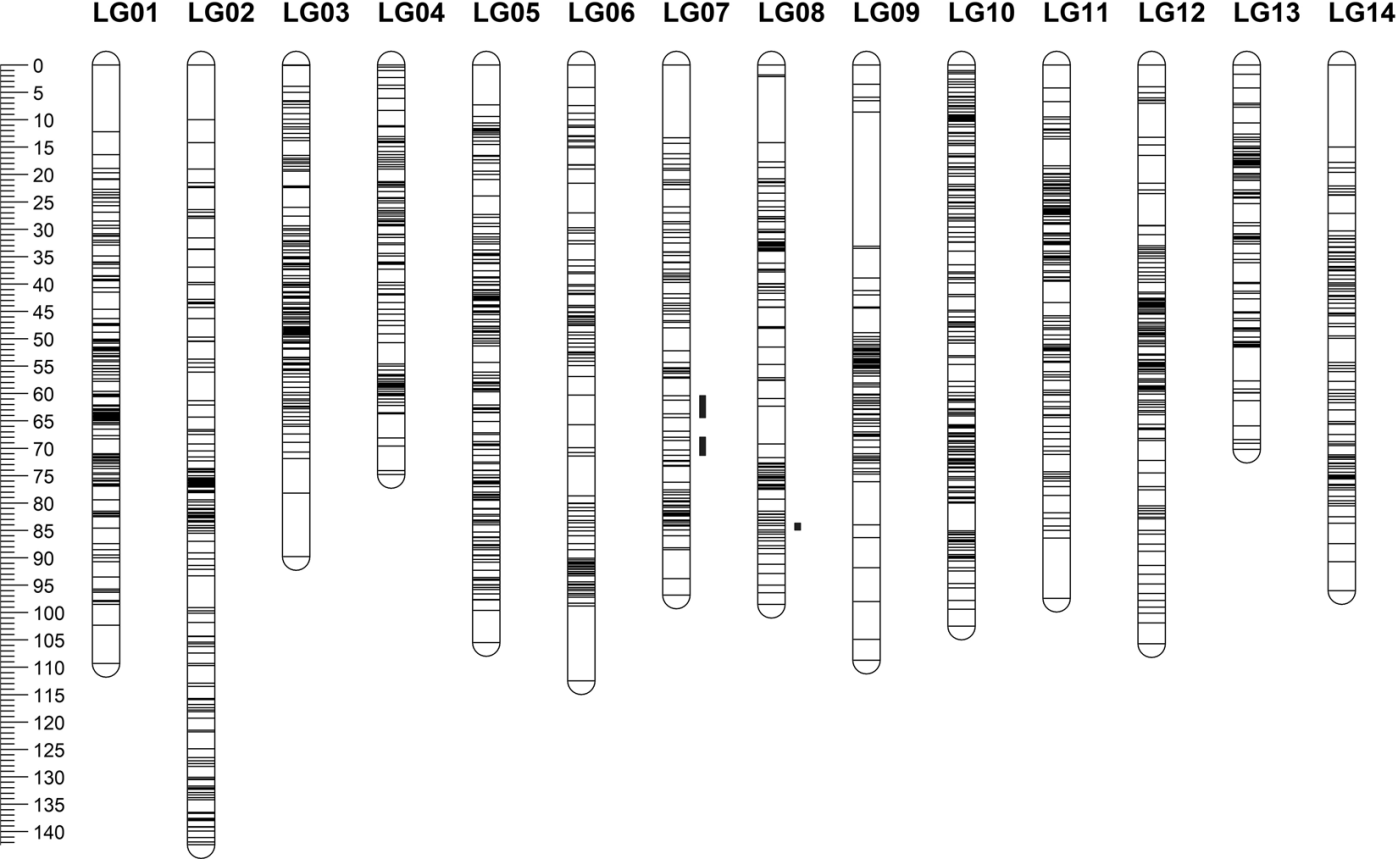
Genotyping by sequencing single nucleotide polymorphism (GBS-SNP) marker distribution for the 14 linkage groups of napiergrass. A black bar means a GBS-SNP marker. A blue bar represents segregation distortion region. The left scale plate represents genetic distance (centiMorgan as unit).

A combined linkage map containing markers that segregated from both female and male parents was constructed, which can facilitate future QTL mapping of the population. To construct a combined linkage map, the markers showing heterozygous on both parents in addition to male-parent heterozygous and female-parent heterozygous markers were used. Therefore, a parent-averaged combined map was constructed by using 378 heterozygous markers for both parents that segregated in a 1:2:1 ratio in the population, in combination with 3,417 male-parent heterozygous and 3,276 female-parent heterozygous markers. In total, 4,058 markers were grouped into 14 linkage groups out of which 1,913 markers were mapped. The final composite linkage map spanned a length of 1,410.10 cM with an average of 0.73 cM between markers. The largest linkage group was Linkage group 02 (LG 02), which spanned 142.40 cM and contained 170 markers (Table 2). Length of each linkage group ranged from 70.18 cM to 142.40 cM and density ranged from 0.88 to 1.77 markers per cM (Fig. 2, Table 2, Supplementary Fig. S8). Results of the χ^2^ test indicated that 114 (6.06%) of the 1,879 markers showed significant segregation distortion (0.001 < P < 0.05) on the combined map. These distorted markers showed clustered distribution on three segregation distortion regions (SDRs) in linkage groups LG07 and LG08 (Fig. 2).

**Table 2:** Summary of the combined linkage map of napiergrass and the percentage of gaps less than 5 cM in male and female parent linkage maps.

Napier grass linkage group	Pearl millet syntenic pseudomolecule	Number of grouped markers	Mapped markers	Unmapped markers	Length (cM)	Density (markers per cM)	Combined map Gaps < = 5 cM (%)	Female parent map Gaps < = 5 cM (%)	Male parent map Gaps < = 5 cM (%)
LG01	PM01	411	163	248	109.33	1.49	98.15	93.98	93.75
LG02	PM06	378	170	208	142.40	1.19	97.63	89.19	92.45
LG03	PM03	360	156	204	89.81	1.74	98.06	96.97	85.71
LG04	PM05	339	127	212	74.83	1.70	99.21	95.51	85.53
LG05	PM02	324	182	142	105.50	1.73	98.34	96.55	96.36
LG06	PM04	300	129	171	112.53	1.15	96.09	96.23	96.83
LG07	PM07	279	99	180	96.84	1.02	96.94	89.8	84.72
LG08	PM06	279	120	159	98.45	1.22	97.48	84	89.86
LG09	PM02	278	96	182	108.70	0.88	92.63	94.64	95.24
LG10	PM01	254	181	73	102.49	1.77	98.89	92.65	75.76
LG11	PM07	237	141	96	97.35	1.45	98.57	91.53	88.06
LG12	PM03	230	146	84	105.72	1.38	97.24	89.29	90.11
LG13	PM05	224	101	123	70.18	1.44	98.00	83.67	94.12
LG14	PM03	165	102	63	96.00	1.06	97.03	89.09	90.91
Total (average)		4,058	1,913	2,145	1,410.10	(1.37)	(97.45)	(91.65)	(89.96)

Among the different reference-based SNP callers, GATK called the highest number of SNPs (197,475) followed by SAMtools and FreeBayes (Table 3). After accounting for segregation ratio and missing data, SAMtools retained the largest number of SNPs followed by TASSEL *de-novo* UNEAK. However, when considering the total number of markers mapped on the combined linkage groups, TASSEL *de-novo* UNEAK showed the highest percentage of SNPs mapped followed by Stacks (Table 3).

**Table 3:** Summary of napiergrass single nucleotide polymorphism (SNP) markers mapped on the combined linkage map using 9 different software pipelines.

Software	Number of SNPs called	Total SNPs used for map construction	No. of SNPs on map	Percentage of SNPs on the map (%)
FreeBayes	25,193	6	0	0.00
GATK	197,475	52	5	0.26
SAMtools	27,082	3,377	151	7.89
GBS-SNP-CROP	2,906	115	52	2.72
TASSEL	7,326	116	56	2.93
Stacks	4,920	447	257	13.43
GBS-SNP-CROP *de-novo*	4,521	96	51	2.67
Stacks *de-novo*	6,871	339	185	9.67
TASSEL *de-novo* UNEAK	10,799	2,523	1,156	60.43
Total	287,093	7,071	1,913	

### Comparison between genomes of napiergrass and pearl millet

**Figure 3:**
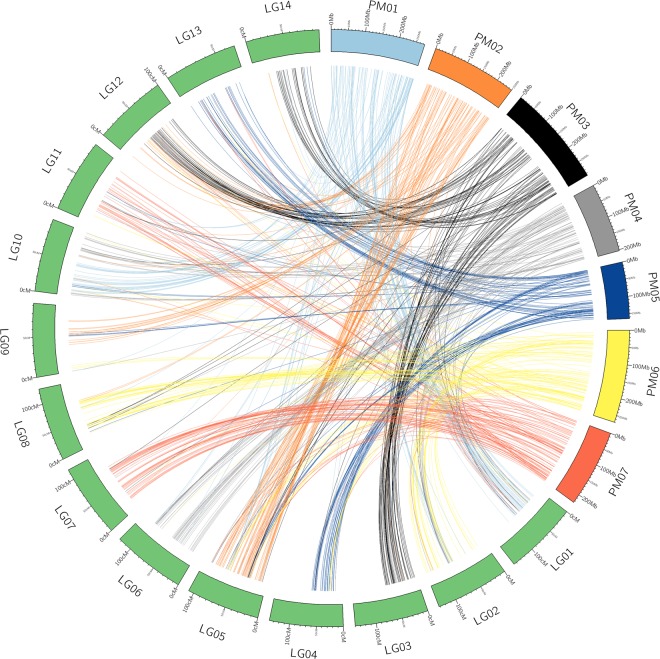
Circos plot of the mapped TASSEL *de-novo* UNEAK napiergrass markers with pearl millet reference genome. Pearl millet pseudomolecules start with “PM” and are color coded for each pseudomolecule. Napiergrass linkage groups start with “LG” and are in green color. Each line corresponds to tags that showed significant BLAST hits to the pearl millet genome (>80% identity and >50 bp length).

**Figure 4:**
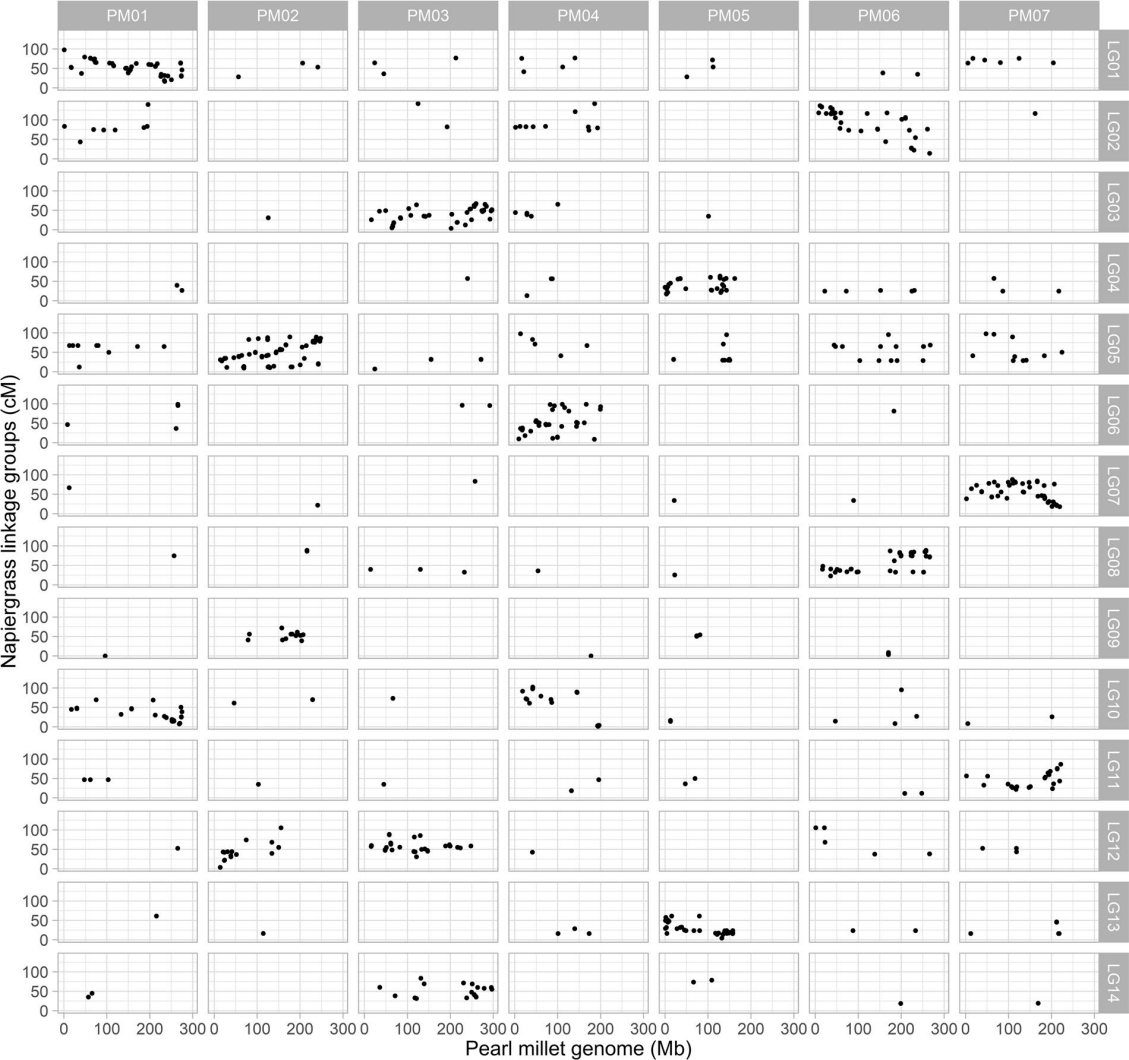
Syntenic regions between napiergrass linkage groups and the pearl millet genome. PM01 to PM07 are pearl millet pseudomolecules, LG01 to LG14 are napiergrass linkage groups. The small dots represent significant BLAST hits of mapped UNEAK tags to the pearl millet genome (>80% identity and >50 bp length).

Sequence tags of the markers that mapped on napiergrass linkage groups were extracted and compared to the pearl millet genome. Among the 1,156 TASSEL *de-novo* UNEAK tags positioned on the combined map, 663 were found to have significant sequence similarities to the genome sequence of pearl millet. Considerable collinearity was observed between the napiergrass and pearl millet genomes (Fig. 3). For each pearl millet pseudomolecule, two corresponding regions in the linkage groups (LGs) of napiergrass genome were identified (Figs 3 and 4). However, some pearl millet genomic regions had more than two corresponding regions on napiergrass genome. For example, pseudomolecule 3 of pearl millet had regions corresponding to three linkage groups LG03, LG12, and LG14 of napiergrass indicating possible chromosomal rearrangement between the two species after speciation (Figs 3 and 4).

## Discussion

Despite its importance as a forage grass and its enormous potential as a biofuel crop, molecular, genetic, and genomic studies have been severely limited in napiergrass. Currently, there was no equivalent genome sequence in the public domain to be used as a reference for napiergrass. In this study, an initial comparison between the napiergrass survey sequences to 10 available grass genomes revealed that napiergrass genomic sequences had the highest similarity with the pearl millet genome, which could be explained by the presence of the A’A’ genome of napiergrass that is homologous to the AA genome of pearl millet. Consequently, in this study we have utilized pearl millet genome v1^40^ as a reference for SNP calling and also performed *de-novo* SNP calling without a reference genome. A total of 38.8% of the napiergrass reads aligned to the pearl millet genome using Bowtie 2, which performed better over BWA, another popular aligner^48,49^. The large portion of unaligned reads might be from the B genome or the divergent chromosome regions of A genome between the two species.

From the genome survey comparison, the total length of all the alignments of napiergrass reads was 25.1% longer in pearl millet indicating genic duplication or expansion in pearl millet and genomic rearrangements between the two species during evolution from their ancestral genome. This is consistent with a previously reported genomic *in situ* hybridization, which verified that the pearl millet genome A was 24% larger compared to the chromosomes of genome A’ of napiergrass^50^. For the 10 longest contigs in our assembly, average repeat content (27%) was lower than reported from other grasses including sorghum (61%)^51^, maize (85%)^52^, foxtail millet (46%)^53^, rice (43.3%)^54^, and pearl millet (77%)^40^. Low repeat content in napiergrass could be attributed to the loss of genomic sequences after hybridization. Rearrangements and loss of genomic sequences are common events after hybridization^55^. Similar to other plant genomes, long-terminal repeat (LTR) retrotransposons comprised the most abundant class (62.19%) of repeats (Supplementary Table S1). Significant relationships between napiergrass, pearl millet, and *P. squamulatum* suggested their common origin and it was inferred that napiergrass and pearl millet had concomitantly diverged from a common ancestor^11,50,56^ and the origin of napiergrass occurred at the interspecific hybridization event, by combining genome A of the ancestor with genome B of a still unknown second ancestor^50^. Our study showed that the napiergrass genome had considerable microcolinearity with the pearl millet genome showing evidence of their relatedness and shared ancestry. Chromosome inverted duplications on pseudomolecule 3 of pearl millet showed possible rearrangement after speciation of napiergrass and pearl millet. Two corresponding regions on the napiergrass linkage groups for each pearl millet chromosome corroborate the hypothesis that these two genomes evolved from a common ancestor.

We developed a limited genomic assembly of napiergrass based on Illumina and 454 sequences. Nearly two thousand SSR markers were developed, which could be immediately useful for applications in napiergrass breeding and genetics. With the advancement of NGS, high throughput NGS-enabled genotyping technologies are becoming readily accessible. Yet, SSR markers remain as a popular tool for genetic studies, variety identification, monitoring of seed purity, and hybrid quality. They are particularly important in laboratories which have limited resources and lack access to NGS facilities or bioinformatic expertise. To our knowledge, this is the first study in napiergrass where SSR markers were developed based on napiergrass genome survey.

A genetic linkage map is an important tool to reveal the genome structure and to identify marker-trait associations^57^ which ultimately help in MAS^33^ to improve precision of selection. In this study, we used the GBS approach to construct a combined high-density linkage map that spanned 1,473.9 cM with 1,917 markers on 14 linkage groups, which is a very critical tool for further genetic and genomics studies of napiergrass. GBS has been extensively used for genotyping many diploid organisms, however, SNP calling from the NGS data in allotetraploids like napiergrass is particularly challenging due to existence of highly similar homeologous copies, one corresponding to A genome and the other to B genome^58^. Therefore, different strategies have been devised to construct linkage map in allopolyploids. For example only uniquely aligned reads (single copy) were considered for SNP calling and subsequent map construction^59,60^ to avoid the collapsed alignment of homoeologous reads due to low divergence, recent polyploidization event, and severe domestic bottlenecks^61^. SNP calling in allotetraploid *Brassica napus* L. (rapeseed; 2n = 4x = 38; AACC) was done by utilizing only uniquely mapped reads (single copy) and a read depth minimum of three to four reads at each potential SNP^59^. Linkage map construction in zoysiagrass (*Zoysia matrella*) was performed by utilizing single-dose markers after calling SNPs using the maximum likelihood method in Stacks^62^. Similarly, single dose markers from TASSEL *de-novo* UNEAK were used to construct linkage maps in prairie cordgrass (*Spartina pectinate*)^63^.

In this study, we applied multiple SNP callers and strategies to maximize SNP calling for linkage map construction for napiergrass. In the final combined genetic map, the number of markers identified by different software varied dramatically. GATK called the highest number of SNPs followed by SAMtools and FreeBayes initially. Both GATK and SAMtools apply Bayesian method to compute the posterior probability for each possible genotype and then choose the genotype with the highest probability as the consensus genotype^64^. GBS-SNP-CROP and TASSEL showed a low matching percentage, which is similar to results from previous research^44^. The number of useful markers for linkage group construction was the highest in SAMtools (47.75%) followed by TASSEL *de-novo* UNEAK (35.68%). However, the TASSEL *de-novo* UNEAK pipeline had the highest number of markers mapped on the linkage groups (60.43%) followed by Stacks (13.43%). This indicated that the network-based SNP discovery in TASSEL *de-novo* UNEAK and UStacks pipeline^65^ could be efficiently utilized for constructing linkage maps in non-model species. Even though TASSEL was primarily designed for diploids, it is powerful enough to give a large number of mapped markers compared to other programs that handle polyploidy like FreeBayes, GATK, or GBS-SNP-CROP.

The SNP markers were relatively evenly distributed among the linkage groups with more than 97.45% of marker interval being less than 5 cM. To our knowledge, this linkage map with an average inter-marker distance of 0.7 cM is the first genetic linkage map of napiergrass to date. A study based on an interspecific population of a cross between pearl millet and napiergrass has been previously reported to link RAPD markers with biomass related traits in *Pennisetum*^23^. The large number of markers and their even distribution in our study facilitate full-scale map coverage. Few regions where the interval space was >5 cM might be due to stretches of large repeats or due to low coverage sequencing of GBS^29,66^. Segregation distortion is regarded as a potential evolutionary force and including these markers for linkage map construction could increase genome coverage as well as benefit QTL mapping^67,68^. Including SDR markers and correcting for bias led to an increase in genetic distance between distorted markers^69^. The deviation from expected Mendelian ratio shows disturbances in the transmission of genetic information from one generation to the next and can be caused by chromosome loss or rearrangements, genetic load, gametic selection, zygotic selection, or both^70–72^. Napiergrass generally outcross through wind pollination that could result in high levels of gene flow leading to genetic load. The assignment of napiergrass linkage groups according to the pearl millet genome allows for future fine mapping and QTL analysis.

In summary, this study reports for the first time a high-density genetic linkage map using NGS-derived SNP markers, as well as the development of SSRs from napiergrass genomic sequences. The napiergrass genome showed considerable collinearity with the pearl millet genome and the genetic map contains 14 linkage groups with low inter-marker interval. The results will be useful for future molecular breeding programs such as identification of QTLs for important traits as well as MAS for the genetic improvement of napiergrass and comparative genomics. These resources will play a critical role in future whole genome sequencing projects and leveraging molecular breeding of napiergrass.

## Methods

### Napiergrass genome survey

The genomic DNA of napiergrass cultivars Merkeron and UF1 was sequenced using Illumina Genome Analyzer and 454 GS-FLX. For Illumina reads, reads that contained more than 50% low-quality bases (Q20) were removed and adapter sequences were trimmed. Quality and adapter trimming of 454 reads was done using default Newbler v2.8 (454 Life Sciences, Roche, Branford, CT) settings. Illumina reads were assembled using ABySS/1.3.4^73^ with kmer size ranging from 25 to 60 at intervals of 5. The 454 reads were assembled using Newbler v2.8 (454 Life Sciences, Roche, Branford, CT) with default parameters. The assemblies were completed using CAP3^74^. The largest 10 contigs of the assembly were selected for further analysis. Repeats on these contigs were masked using a comprehensive public repeat database compiled from TIGR plant repeats (http://plantrepeats.plantbiology.msu.edu/), Plant miniature inverted-repeat transposable elements (P-MITE) database (http://pmite.hzau.edu.cn/django/mite/), MIPS Repeat Element Database (http://mips.helmholtz-muenchen.de/plant/recat/), and Repbase from RepeatMasker software (http://www.repeatmasker.org/). Unique repeats were extracted from this database by removing redundant repeats with 98% identity using CD-HIT/4.6^46^. SNPs and indels were called using FreeBayes/0.9.15^46^ excluding alleles with depth less than 20. The annotation of SNPs was performed using SnpEff/4.0 (http://snpeff.sourceforge.net/)^75^. In order to identify sequence similarity among the two genomes, clean reads from Illumina and 454 were aligned to the pearl millet genome v1^40^ using bowtie2/2.2.5.

### SSR identification and marker development

The napiergrass assembly was used to identify SSR markers that contained repeat motifs ranging in length from 1 to 6 bp. The minimum number of repeats was 10 for Mono-, 6 for Di-and 5 for Tri-, Tetra-, Penta- and Hexa-. SSRs were analyzed based on their types, number of repeats, and percentage frequency of occurrences of each SSR motif. SSRs in napiergrass were detected using MIcroSAtellite identification tool (MISA)^76^ and primers were developed using primer3 software^77^. SSR search results were input into scripts p3_in.pl and p3_out.pl in order to identify SSR primer pairs for napiergrass.

### Plant materials and DNA extraction

A mapping population of 185 F_1_ hybrid progenies were developed from a cross between two napiergrass accessions (N122 and N190) described previously^41,78^. The 185 F_1_ hybrids were planted in the field at the Plant Science Research and Education Unit (PSREU), Citra, Florida, along with the parental accessions.

Young and healthy leaf tissues were harvested from each individual of the mapping population. DNA extraction was done following the protocol described previously^79^. The extracted DNA samples were run on a 2% agarose gel to check the quality and quantified with PicoGreen to meet the requirements of GBS. 185 F_1_ plants that were confirmed to be true hybrids using SSR markers^41^ were selected for GBS.

### Genotyping by sequencing

GBS data was generated at the Institute of Biotechnology, Cornell University as described previously^30^. Briefly, DNA samples were digested with the restriction enzyme *Pst*I followed by ligation of adapters, that consisted of Illumina sequencing primers and barcode adapters, to the DNA fragment ends. After ligation, 95 samples were combined into a pool and PCR amplification was performed to create a GBS library and sequenced on Illumina HiSeq 2000.

### Comparative genomics

Unique tags of napiergrass from TASSEL *de-novo* UNEAK/3.0^42^ pipeline were used for comparative genomic analysis. CD-HIT/4.6.4^80^ was used to cluster the tags. Genomes of rice (Osativa_323_v7.0), *Brachypodium* (Bdistachyon_314_v3.0), maize (Zmays_284_AGPv3), sorghum (Sbicolor_313_v3.0), foxtail millet (Sitalica_312_v2), switchgrass (Pvirgatum_273_v1.0), wheat (Taestivum_296_v2), *Arabidopsis* (Athaliana_167_TAIR9) were downloaded from Phytozome v11 (https://phytozome.jgi.doe.gov/pz/portal.html). The barley genome (ASM32608v1) was downloaded from Ensembl (http://www.ensembl.org). We used BLASTN (BLAST v2.5.0) with the default settings and an e-value cutoff of 1 × 10^−8^ to blast the uniquely clustered tags of napiergrass against different genomes in order to find the percentage similarity of napiergrass reads among the various grass species. Tags of 64 bp with >80% identity and alignment length >50 bp to the respective genomes were counted as a hit.

### Sequence analysis and SNP calling

Raw data processing and SNP identification was performed using both *de novo* and reference-based approaches. Common software capable of calling SNPs *de novo* used in this research were TASSEL/3.0 UNEAK^42^, Stacks/1.24^81^, and GBS-SNP-CROP 1.1^44^. For the reference based approach, pearl millet reference genome v1^40^ was used. The reference genome consists of seven pseudomolecules. Six different reference based pipelines were evaluated to call SNPs viz., TASSEL 4.3^42^, Stacks 1.24^43^, GBS-SNP-CROP 1.1^44^, SAMtools 1.2 mpileup^45^, FreeBayes 0.9.21^46^, and GATK 3.3^47^. Parameters used in each software were provided in Supplementary Table S6. Sequence variants called were filtered with a minimum depth of 48 per sample.

### Linkage map construction

QC-filtered SNPs were further filtered by the following standards for map construction: (1) markers must be genotyped in at least 180 individuals; (2) Individuals with over 10% missing data were discarded; and (3) Redundant markers were removed by standard of similarity = 1. For each parental map construction, only single dose markers were used. Markers segregating at a distorted Mendelian ratio (expected ratio for ‘lmxll’ type and ‘nnxnp’ type is 1: 1, χ2 test, 0.001 < P < 0.05) were marked. The single dose markers from the maternal and paternal parent were analyzed separately using JoinMap 4.1^82^ and outcross pollinated family (CP) was selected as the population type. Markers that were heterozygous in N122 and homozygous in N190 (‘lmxll’ type) were selected to build N122 linkage groups. Markers that were heterozygous in N190 and homozygous in N122 (‘nnxnp’ type) were selected to build N190 linkage groups. The linkage groups were built using regression mapping algorithm, with a minimum logarithm of odds (LOD) value at 20, and a maximum recombination frequency at 0.40. Marker positioning calculation was performed with a goodness-of-fit jump at 5, followed by a “ripple” procedure (value = 1). Map distances were estimated using the Kosambi mapping function. Genetic distance between SDR markers were corrected using DistortedMap^69^. Linkage maps were drawn with MapChart^83^. For integrated map construction, markers that were heterozygous in both parents (‘hkxhk’ type) were selected to build combined linkage groups. Markers segregating at distorted Mendelian ratio (expected ratio for ‘hkxhk’ type is 1:2:1, χ2 test, 0.001 < P < 0.05) were marked. The retained markers were then added with the markers from male and female parents to construct a combined map. The linkage groups were built using regression mapping algorithm, with a minimum logarithm of odds (LOD) value at 20, and a maximum recombination frequency of 0.40. Other parameters were the same with linkage map construction above. Regions showing segregation distortion (0.001 < P < 0.05) with more than three adjacent loci were marked as SDR regions^84,85^.

### Comparison between napiergrass and pearl millet genome

Consensus sequence of mapped markers from TASSEL *de-novo* UNEAK were used to compare with the reference genome of pearl millet with same parameters (BLASTN defaults with an e-value cutoff of 1 × 10^−8^). Markers that showed significant hits to the genome sequence and/or gene models of pearl millet with >80% identity and alignment length >50 bp were extracted and used for comparative genomics study. A circos plot was drawn using circos/0.69-2^86^.

## Electronic supplementary material

Supplementary Tables and Figures

Supplementary Tables S3 and S4

## Data Availability

All raw data from the genotyping-by-sequencing runs is deposited at NCBI on PRJNA380523.
