# Supplementary material for: Surveying the genome and constructing a high-density genetic map of napiergrass (Cenchrus purpureus Schumach)
Source: Sci Rep. 2018 Sep 26;8:14419. doi: 10.1038/s41598-018-32674-x (PMC6158254; doi:10.1038/s41598-018-32674-x)
Supplement: Supplementary file 1 — Supplementary Tables and Figures [file 41598_2018_32674_MOESM1_ESM.docx]

**Title: Surveying the genome and constructing a high-density genetic map of napiergrass (*Cenchrus purpureus* Schumach.)**

**Authors: Dev Paudel, Baskaran Kannan, Xiping Yang, Karen Harris-Shultz, Mahendar Thudi, Rajeev K. Varshney, Fredy Altpeter, Jianping Wang***

**SUPPLEMENTARY FILES**

**Legend:**

# Supplementary Tables

Supplementary Table S1. Repetitive elements present in the napiergrass genome

Supplementary Table S2: The sequence alignment of ten napiergrass sequence contigs to the pearl millet genome

Supplementary Table S3. Frequency of classified repeat types (considering complementarity) in napiergrass

Supplementary Table S4. Primer pairs developed for napiergrass SSR markers

Supplementary Table S5. Alignment of individual napiergrass reads using Bowtie2

Supplementary Table S6. Parameters used for SNP calling for each software

# Supplementary figures

Supplementary Figure S1. Sequence variation for SNPs called in various regions of the pearl millet genome

Supplementary Figure S2. Micro-collinearity between contigs from napiergrass to the pearl millet genome

Supplementary Figure S3. Inversion duplication between napiergrass and pearl millet

Supplementary Figure S4: Estimated coverage of *Pst*I restriction sites in the pearl millet genome

Supplementary Figure S5. Histogram of uniquely mapped reads to the pearl millet genome

Supplementary Figure S6. Genetic linkage map of the napiergrass female parent N190

Supplementary Figure S7. Genetic linkage map of the napiergrass male parent N122

Supplementary Figure S8. Consensus genetic linkage map of napiergrass

**SUPPLEMENTARY TABLES**

**Supplementary Table S1. Repetitive elements present in the napiergrass genome**

| Transposable Element | | Count |
| --- | --- | --- |
| DNA transposon |  |  |
|  | Tc1/Mariner | 8 |
|  | hAT | 20 |
|  | PIF/Harbinger | 16 |
|  | EnSpm | 1 |
|  | CACTA | 4 |
|  | Polinton | 1 |
| LTR Retrotransposon | |  |
|  | LTR | 37 |
|  | LTR/Copia | 5 |
|  | LTR/Gypsy | 5 |
|  | Copia | 4 |
|  | Retrotransposon | 3 |
|  | Retroelement | 1 |
|  | Gypsy | 2 |
| Non-LTR Retrotransposons | |  |
|  | L1 | 2 |
| Pseudogene |  |  |
|  | tRNA | 1 |
|  | rRNA | 8 |
|  | rDNA-like | 3 |
| Others |  |  |
|  | Mutator | 15 |
|  | Telomeric | 4 |
|  | MobileElement | 2 |
|  | Micro-like sequence | 1 |
|  | Low-complexity | 3 |
|  | Simple Repeats | 12 |
|  | Unspecified | 6 |
| Total |  | 164 |

**Supplementary Table S2: The sequence alignment of ten napiergrass sequence contigs to the pearl millet genome**

| **No.** | **Napiergrass contig** | **Size of contig (bp)** | **GC content (%)** | **Repeat content (%)** | **Corresponding pearl millet pseudomolecule** | **Number of hits above 500bp** | **Number of predicted genes** | **Alignment length in napiergrass** | **Alignment length in pearl millet** | **Sum of expanded length in pearl millet** |
| --- | --- | --- | --- | --- | --- | --- | --- | --- | --- | --- |
| 1 | Contig1 | 8,506 | 55.3 | 80.3 | Pg5 | 0 | 0 | / | / | / |
| 2 | Contig3434 | 9,653 | 42.5 | 9.8 | Pg3 | 5 | 0 | 5,407 | 5,384 | -23 |
| 3 | Contig5516 | 11,920 | 44.9 | 17.5 | Pg3 | 11 | 1 | 6,776 | 13,509 | 6733 |
| 4 | Contig5578 | 8,558 | 42.8 | 5.9 | Pg2 | 3 | 0 | 5,948 | 6,487 | 539 |
| 5 | Contig5588 | 8,595 | 44.6 | 14 | C26927002 | 1 | 1 | 591 | 591 | 0 |
| 6 | Contig5729 | 9,088 | 46 | 20.4 | Pg5 | 3 | 0 | 2,250 | 2,246 | -4 |
| 7 | Contig5798 | 8,651 | 43.1 | 5.3 | C27370090 | 1 | 0 | 1,385 | 1,385 | 0 |
| 8 | Contig5878 | 25,329 | 43.4 | 12.8 | Pg6 | 4 | 0 | 5,692 | 5,676 | -16 |
| 9 | Contig5902 | 13,801 | 46.1 | 16.4 | Pg6 | 8 | 0 | 6,209 | 7,596 | 1387 |
| 10 | Contig6139 | 14,330 | 42.2 | 93.1 | Pg7 | 0 | 0 | / | / | / |
| **Total** | **10** | **118,431** | **45.09** | **27.5** | **8** | **36** | **2** | **34,258** | **42,874** | **8,616** |

**Supplementary Table S5. Alignment of individual napiergrass reads using Bowtie2**

|  | Number of raw reads | Clean reads (retained by Stacks) | % of reads retained | Total reads mapped to pearl millet | Uniquely mapped | % uniquely mapped |
| --- | --- | --- | --- | --- | --- | --- |
| Min. | 44,858 | 20,350 | 45.37% | 16,658 | 1,140 | 6.00% |
| Max. | 5,046,114 | 4,739,605 | 97.53% | 3,022,185 | 1,902,058 | 45.00% |
| Avg. | 2,893,836.07 | 2,696,528.11 | 92.71% | 1,727,194.04 | 1,077,802.96 | 39.68% |
| Total | 549,828,854 | 512,340,341 | 93.18% | 328,166,867 | 204,782,562 | 39.97% |

**Supplementary Table S6. Parameters used for SNP calling for each software**

| **Reference based** | **Parameters** | **Remarks. [defaults]** |
| --- | --- | --- |
| TASSEL 4.3 | -c 5 | Min. number of times a tag must be present to be output <5> [1] |
|  | -mnMAF 0.01 | Min. minor allele frequency <0.01>[0.01] |
|  | -mnMAC 100000 | Min. minor allele count <100000>[10] (SNPs that pass either -mnMAF or -mnMAC will be output) |
|  | -misMat 2 | Threshold genotypic mismatch rate above which the duplicate SNPs won’t be merged <2>[0.05] |
|  | -callHets | When two genotypes at a replicate SNP disagree for a taxon, call it a heterozygote |
| Stacks | -A CP | CP type for genetic map |
|  | -m 3 | Min. number of identical, raw reads required to create a stack <3>[3] |
| GBS-SNP-CROP | -l 30  -sl 4:30  -tr 30  -m 32 | Trimmomatic LEADING parameter  Trimmomatic SLIDINGWINDOW parameter  Trimmomatic TRAILING parameter  Trimmomatic MINLEN parameter |
|  | -rl 100  -pl 32  -p 0.01  -id 0.93 | Raw GBS read length  Min. length required after merging to retain read  p-value for PEAR  Nucleotide identity value required for USEARCH read clustering |
|  | -Q 30  -q 0  -f 0  -F 2308 | Phred score base call quality  Alignment quality  SAMtools flags  SAMtools flags |
|  | -mnHoDepth0 11 | Min. depth required for calling a homozygote when the alternative allele depth = 0 |
|  | -mnHoDepth1 48 | Min. depth required for each allele when calling a heterozygote |
|  | -mnHetDepth 3 | Min. depth required for each allele when calling a heterozygote |
|  | -altStrength 0.9 | Across the pop. For a given putative bi-allelic SNP, this alternate allele strength is the minimum proportion of non-primary allele reads that are the secondary allele |
|  | -mnAlleleRatio 0.1 | Min. required ratio of less frequent allele depth to more frequent allele depth |
|  | -mnCal 0.75 | Min. acceptable proportion of genotyped individuals to retain a SNP |
|  | -mnAvgDepth 4 | Min. avg. depth of an acceptable SNP |
|  | -mxAvgDepth 200 | Max avg. depth of an acceptable SNP |
| SAMtools mpileup | -uf | Default |
| FreeBayes | -C 2 | --min-alternate-count Require at least <2> observations supporting an alternate allele within a single individual in order to evaluate the position [1] |
|  | -p 4 | --ploidy <4> [2] |
|  | --use-best-n-alleles 4 | Evaluate only the best N SNP alleles ranked by sum of supporting quality scores [all] |
|  | --min-coverage 5 | Require at least <5> coverage to process a site [0] |
| GATK | -T UnifiedGenotyper | Call SNPs and indels on a per-locus basis |
|  | -stand_call_conf 30 | The min. phred-scaled confidence thresholds at which variants should be called <30> [30] |
|  | -stand_emit_conf 10 | The minimum phred-scale confidence threshold at which variants should be emitted (and filtered with LowQual if less than the calling threshold) <10> [30] |
|  | -ploidy 4 | Ploidy <4> [2] |
|  | -mbq 20 | Minimum base quality required to consider a base for calling |
|  | -glm BOTH | Genotype likelihoods calculation model <BOTH> includes SNPs and INDELs [SNP] |

| **de-novo based** | **Parameters** | **Remarks. defaults []** |
| --- | --- | --- |
| UNEAK | -e PstI | Restriction enzyme used |
|  | -c 5 | Min. count of a tag must be present to be output [5] |
|  | -e 0.03 | Error tolerance rate in the network filter [0.03] |
|  | -mnMAF 0.05 | Min. minor allele frequency [0.05] |
|  | -mxMAF 0.5 | Max. minor allele frequency [0.5] |
|  | -mnC 0 | Min. call rate (proportion that how many taxa are covered by at least one tag) |
|  | -mxC 1 | Max. call rate [1] |
| Stacks | -m 3 | Min. number of identical, raw reads to create a stack |
|  | -M 2 | No. of mismatches allowed between loci when processing a single individual [2] |
|  | -n 1 | No. of mismatches allowed between loci when building the catalog [1] |
|  | -t | Remove, or break up, highly repetitive RAD-Tags in the ustacks program |
| GBS-SNP-CROP |  | Same as reference based except, script 3 settings not required |

**SUPPLEMENTARY FIGURES**

**Supplementary Figure S1. Sequence variation for SNPs called in various regions of the pearl millet genome**


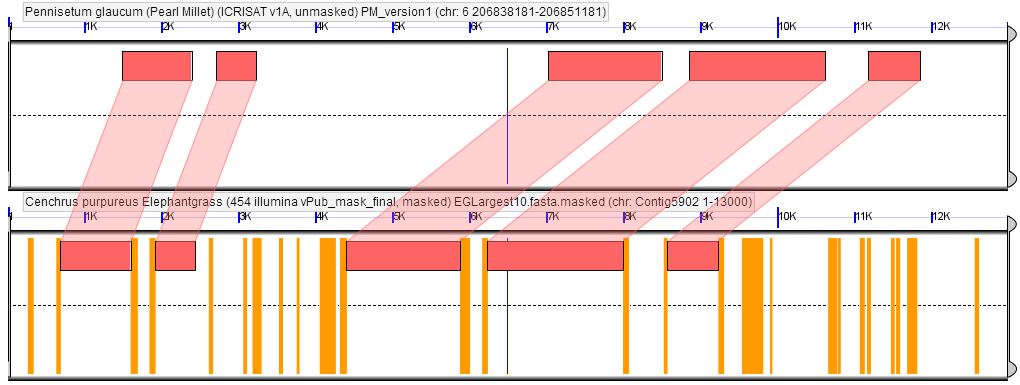


**Supplementary Figure S2. Micro-collinearity between contigs from napiergrass to the pearl millet genome**


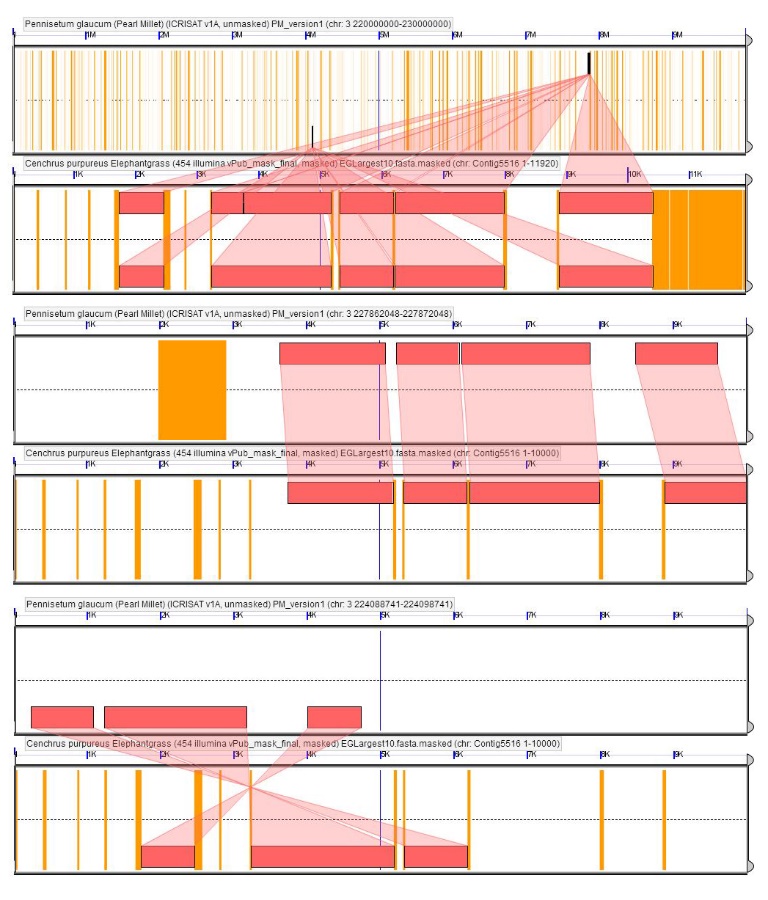
 **Supplementary Figure S3. Inversion duplication between napiergrass and pearl millet (shown in bottom figure)**

**Supplementary Figure S4: Estimated coverage of *Pst*I restriction sites in the pearl millet genome**

**Supplementary Figure S5. Histogram of uniquely mapped reads to the pearl millet genome**

**Supplementary Figure S6. Genetic linkage map of the napiergrass female parent N190**

**Supplementary Figure S7. Genetic linkage map of the napiergrass male parent N122**

**Supplementary Figure S8. Consensus genetic linkage map of napiergrass**
